# phoenix: an R package and Python module for calculating the Phoenix pediatric sepsis score and criteria

**DOI:** 10.1093/jamiaopen/ooae066

**Published:** 2024-07-04

**Authors:** Peter E DeWitt, Seth Russell, Margaret N Rebull, L Nelson Sanchez-Pinto, Tellen D Bennett

**Affiliations:** Department of Biomedical Informatics, University of Colorado School of Medicine, University of Colorado Anschutz Medical Campus, Aurora, CO 80045, United States; Department of Biomedical Informatics, University of Colorado School of Medicine, University of Colorado Anschutz Medical Campus, Aurora, CO 80045, United States; Department of Biomedical Informatics, University of Colorado School of Medicine, University of Colorado Anschutz Medical Campus, Aurora, CO 80045, United States; Department of Pediatrics (Critical Care), Northwestern University Feinberg School of Medicine, and Ann and Robert H. Lurie Children’s Hospital of Chicago, Chicago, IL 60611, United States; Department of Preventive Medicine (Health and Biomedical Informatics), Northwestern University Feinberg School of Medicine, and Ann and Robert H. Lurie Children’s Hospital of Chicago, Chicago, IL 60611, United States; Department of Biomedical Informatics, University of Colorado School of Medicine, University of Colorado Anschutz Medical Campus, Aurora, CO 80045, United States; Section of Critical Care Medicine, Department of Pediatrics, University of Colorado School of Medicine, University of Colorado Anschutz Medical Campus, Aurora, CO 80045, United States

**Keywords:** sepsis, septic shock, pediatrics, computer software, EHR

## Abstract

**Objectives:**

The publication of the Phoenix criteria for pediatric sepsis and septic shock initiates a new era in clinical care and research of pediatric sepsis. Tools to consistently and accurately apply the Phoenix criteria to electronic health records (EHRs) is one part of building a robust and internally consistent body of research across multiple research groups and datasets.

**Materials and Methods:**

We developed the phoenix R package and Python module to provide researchers with intuitive and simple functions to apply the Phoenix criteria to EHR data.

**Results:**

The phoenix R package and Python module enable researchers to apply the Phoenix criteria to EHR datasets and derive the relevant indicators, total scores, and sub-scores.

**Discussion:**

The transition to the Phoenix criteria marks a major change in the conceptual definition of pediatric sepsis. Applicable across differentially resourced settings, the Phoenix criteria should help improve clinical care and research.

**Conclusion:**

The phoenix R package and Python model are freely available on CRAN, PyPi, and GitHub. These tools enable the consistent and accurate application of the Phoenix criteria to EHR datasets.

## Introduction

Approximately 3.3 million pediatric deaths per year are attributable to sepsis and septic shock.^1^ In January 2024, the Phoenix diagnostic criteria for pediatric sepsis were published to supersede the criteria^2^ defined by the International Pediatrics Sepsis Criteria Conference in 2005.^3^^,^^4^ Transitioning to the Phoenix criteria is a conceptual change, moving away from an inflammatory response-based criteria to a life-threatening organ dysfunction-based criteria. This change parallels the conceptual change for the diagnostic criteria of adult sepsis.^5^^,^^6^

The Pediatric Sepsis Task Force developed the Phoenix criteria using a data-driven modified Delphi consensus approach. The criteria are based on 4 organ dysfunctions: respiratory, cardiovascular, coagulation, and neurologic. Additionally, the task force published an 8-organ system score, Phoenix-8, for research purposes.^3^^,^^4^

The Phoenix criteria initiate a new era of benchmarking, epidemiological surveillance, clinical quality improvement, and research in pediatric sepsis.

Sufficient information to implement the Phoenix criteria has been published.^3^^,^^4^ However, the published code required extensive redactions to protect the anonymity of both patients and health care systems providing data, resulting in code that is not easily reusable.

There is a need for a tool that can apply the Phoenix criteria to any electronic health record (EHR) dataset consistently such that publications reporting on or using the Phoenix criteria can be compared fairly among each other.

To fill this need, we have developed and published a R package, Python module, and example SQL queries for applying the Phoenix criteria to other datasets.

## Objectives

To provide an efficient and consistent way to apply the Phoenix scoring rubric to new EHR datasets and for all researchers, we developed the phoenix R package, Python module, and example SQL queries.

## Methods

The Phoenix criteria (Table 1) were developed using a dataset that included over 3.5 million pediatric encounters from 10 hospital systems across North America, South America, Asia, and Africa. The criteria are applicable to pediatric patients in both high- and low-/middle-resourced environments. The development data excluded birth-hospitalizations and patients with gestational ages less than 37 weeks.^3^^,^^4^

**Table 1.** The organ dysfunction scoring for the Phoenix criteria.

Organ system	0 Points	1 Point	2 Points	3 Points
**Respiratory** (0-3 points)				
Respiratory support		Any respiratory support	IMV^a^	IMV
PaO_2_: FiO_2_	≥ 400	< 400	< 200	< 100
SpO_2_: FiO_2_^b^	≥ 292	< 292	< 220	< 148
**Cardiovascular** (0-6 points; sum of medications, lactate, and MAP)				
Systemic vasoactive medications^c^	No medications	1 medication	2 or more medications	
Lactate^d^ (mmol/L)	< 5	5 ≤ lactate^e^ < 11	≥ 11	
**Age** ^f^ (months) adjusted MAP^g^ (mmHg)				
0 ≤ Age < 1	≥ 31	17 ≤ MAP < 31	< 17	
1 ≤ Age < 12	≥ 39	25 ≤ MAP < 39	< 25	
12 ≤ Age < 24	≥ 44	31 ≤ MAP < 44	< 31	
24 ≤ Age < 60	≥ 45	32 ≤ MAP < 45	< 32	
60 ≤ Age < 144	≥ 49	36 ≤ MAP < 49	< 36	
144 ≤ Age < 216	≥ 52	38 ≤ MAP < 52	< 38	
**Coagulation** ^h^ (0-2 points; 1 for each lab; max of 2 points)				
Platelets (1000/μL)	≥ 100	< 100		
INR	≤ 1.3	> 1.3		
D-dimer (mg/L FEU)	≤ 2	> 2		
Fibrinogen (mg/dL)	≥ 100	< 100		
**Neurologic** ^i^ (0-2 points)				
	GCS^j^ ≥ 11	GCS ≤ 10	Bilaterally fixed pupils	
**Endocrine** (0-1 point)				
Blood glucose (mg/dL)	50 ≤ Blood Glucose ≤ 150	< 50; or > 150		
**Immunologic** (0-1 point; point from ANC and/or ALC)				
ANC (cells/mm3)	≥ 500	< 500		
ALC (cells/mm3)	≥ 1000	< 1000		
**Renal** (0-1 point)				
**Age** ^k^ (months) adjusted Creatinine (mg/dL)				
0 ≤ Age < 1	< 0.8	≥ 0.8		
1 ≤ Age < 12	< 0.3	≥ 0.3		
12 ≤ Age < 24	< 0.4	≥ 0.4		
24 ≤ Age < 60	< 0.6	≥ 0.6		
60 ≤ Age < 144	< 0.7	≥ 0.7		
144 ≤ Age < 216	< 1.0	≥ 1.0		
**Hepatic** (0-1 point; point from total bilirubin and/or ALT)				
Total bilirubin (mg/dL)	< 4	≥ 4		
ALT (IU/L)	≤ 102	> 102		

The Phoenix sepsis criteria are based on the Phoenix Sepsis Score, which includes respiratory, cardiovascular, coagulation, and neurologic dysfunction; Phoenix-8 is based on those 4 organ systems plus endocrine, immunologic, renal, and hepatic dysfunction. Sepsis is defined as a Phoenix Sepsis Score ≥ 2. Septic shock is defined as sepsis with at least one cardiovascular point. Missing data maps to scores of zero. The limits reported in this table reflect the implementation of the criteria in software, whereas the comparable published tables report the criteria from a clinical perspective.^3^^,^^4^ The 2 representations of the criteria are consistent in practice.

a ALC, Absolute lymphocyte count; ALT, alanine aminotransferase; ANC, Absolute neutrophil count; FEU, fibrinogen equivalent units; FiO_2_, fraction of inspired oxygen; GCS, Glasgow Coma Score; IMV, invasive mechanical ventilation; INR, International normalized ratio; MAP, mean arterial pressure; PaO_2_, arterial oxygen pressure; SpO_2_, pulse oximetry oxygen saturation.

b SpO_2_: FiO_2_ is only valid when SpO_2_ ≤ 97.

c Vasoactive medications: any systemic dose of dobutamine, dopamine, epinephrine, milrinone, norepinephrine, and/or vasopressin.

d Lactate can be arterial or venous. Reference range 0.5-2.2 mmol/L.

e The verbosity of this table is greater than in the tables in the original source publications.^3^^,^^4^ The inequalities reported in this table, and the specific values reported in this table, reflect how the criteria is implemented in software whereas the source publications reported tables consistent with clinical practice. A couple notable differences. 1 cardiovascular point is reached for a lactate value of “5-10.9 mmol/L” and 2 points for lactate ≥ 11 mmol/L.^3^^,^^4^ There is an implication of rounding lactate to 1 decimal place and assessing the criteria. The software simplifies the work by considering lactate values to be a floating point value that could take on any real value and thus the logic of “5 ≤ lactate < 11” for 1 point. Additionally, for MAP, the criteria listed in this table is consistent with common clinical practice of interpreting MAP as integer values. The criteria listed in this table is used with the assumption that MAP values are floating point values.

f Age: measured in months and is not adjusted for prematurity.

g MAP: Use measured mean arterial pressure preferentially (invasive arterial if available, or non-invasive oscillometric), alternatively use the calculation diastolic + (systolic—diastolic)/3.

h Coagulation variable reference ranges: platelets, 150-450 103/μL; D-dimer, < 0.5 mg/L FEU; fibrinogen, 180-410 mg/dL. International normalized ratio reference range is based on local reference prothrombin time.

i Neurologic dysfunction scoring was pragmatically validated in both sedated and on sedated patients and those with and without IMV.

j GCS measures level of consciousness based on verbal, eye, and motor response. Values are integers from 3 to 15 with higher scores indicating better neurologic function.

k Age: measured in months and is not adjusted for prematurity.

The Phoenix criteria define sepsis as a suspected infection (operationalized as at least one dose of a systemic anti-microbial medication and at least one microbiological test ordered within the first 24 hours of a hospital encounter) with a Phoenix score of at least 2 points. Additionally, septic shock is defined as sepsis with at least one point from the cardiovascular dysfunction component of the Phoenix Sepsis Score.^3^^,^^4^

Missing data values are mapped to scores of zero.^4^ It was reasonable to assume that for some laboratory values and metrics missing data indicate no concern and testing was not ordered. Furthermore, the Phoenix criteria was developed to be useful in high-, medium-, and low-resource settings where some laboratory values, medications, and other values might be uncommon or impossible to obtain. The phoenix R package, Python module, and example SQL queries handle missing values consistent with the development approach for the Phoenix criteria.

The phoenix R package is available from the Comprehensive R Archive Network (CRAN) (https://cran.r-project.org/package=phoenix) and GitHub (https://github.com/cu-dbmi-peds/phoenix/). phoenix was designed to be as light-weight as possible. There are no dependencies nor imports save base R. The R package was the primary focus for development and will be the focus for this manuscript. A testing suite for version 1.0.0 of phoenix has 100% code coverage (details on the GitHub page) along with automatic CRAN checks for Windows, MacOS, and Ubuntu, for the current version of R, the prior version of R, and the development version of R.

The Python module has been made public via PyPi (https://pypi.org/project/phoenix-sepsis/) with source code available in the same GitHub repository as the R package. A set of tests are built within the GitHub source code to ensure that the results of the Python module are identical to the results of the R package.

Lastly, example SQL queries, as understood by SQLite, are also provided in the GitHub repository and the Supplementary Examples. As with the Python module, there is testing code within the repo to ensure the results of the SQLite queries are identical to the R package.

Extensive documentation for the R package, Python module, and SQLite queries are available on the package website (https://cu-dbmi-peds.github.io/phoenix/).

## Results

An example dataset, sepsis, is provided as a data.frame within the R package, as a plain text file in the Python module, and used in the example SQLite queries. The data consist of 20 synthetic observations of 27 variables needed by the Phoenix and Phoenix-8 criteria. The data are lazyloaded in R and are available when the phoenix namespace is attached to the search path, that is, when library(phoenix) is called.


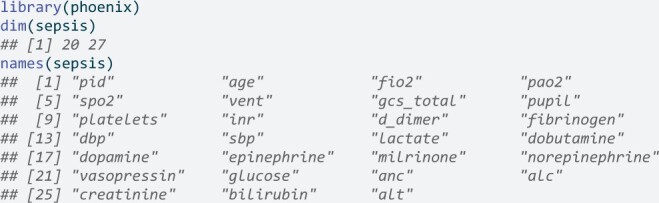


In Python, the example data can be loaded into a pandas DataFrame via


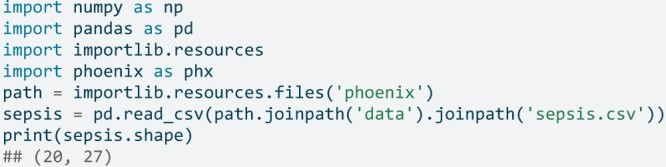


Extensive detail on the synthetic data is available in the R package documentation, the package website, and in the Supplementary Examples.

End users of the Python module and R package will have generally the same experience. Both use the same naming conventions and provide the same 10 vectorized functions for applying the Phoenix criteria (Table 2).

**Table 2.** R and Python function names and returns for each of the Phoenix organ dysfunction scores and total scoring.

Phoenix criteria	R, Python function	R return	Python return
Respiratory dysfunction	phoenix_respiratory	Integer vector	Numpy array
Cardiovascular dysfunction	phoenix_cardiovascular	Integer vector	Numpy array
Coagulation dysfunction	phoenix_coagulation	Integer vector	Numpy array
Neurologic dysfunction	phoenix_neurologic	Integer vector	Numpy array
Endocrine dysfunction	phoenix_endocrine	Integer vector	Numpy array
Immunologic dysfunction	phoenix_immunologic	Integer vector	Numpy array
Renal dysfunction	phoenix_renal	Integer vector	Numpy array
Hepatic dysfunction	phoenix_hepatic	Integer vector	Numpy array
Phoenix criteria	phoenix	data.frame	Pandas DataFrame
Phoenix-8 criteria	phoenix8	data.frame	Pandas DataFrame

The Phoenix criteria is the sum of the respiratory, cardiovascular, coagulation, and neurologic scores. Phoenix sepsis is defined as a total score of 2 or more points (along with suspected infection). Septic shock is sepsis with at least one cardiovascular point. Phoenix-8 is an extended scoring system and is the sum of all 8 organ systems.

The return of phoenix() is a data.frame (R) Pandas DataFrame (Python) with 7 columns; the respiratory dysfunction score, cardiovascular dysfunction score, coagulation dysfunction score, neurologic dysfunction score, total score, and indicator columns for sepsis (total score ≥ 2), and septic shock (sepsis with ≥ 1 cardiovascular points). phoenix8() returns the same as phoenix() with additional columns for the endocrine, immunologic, renal, and hepatic dysfunction scores, and the Phoenix-8 total score. All the columns are integer valued.

A simple example^4^: a 3-year-old presenting with a fever, tachycardia, and irritability is given broad spectrum antibiotics and started on a norepinephrine drip due to hypotension (blood pressure 67/32). A complete blood count (CBC) shows a platelet count of 95 K/μL. Applying the Phoenix criteria to this patient results in a respiratory score of 0, cardiovascular score of 2; coagulation score of 1; neurologic score of 0, and a total score of 3; sepsis=yes because the score is ≥ 2; septic shock=yes because the cardiovascular score is also ≥ 1. Scoring in R would be done using:


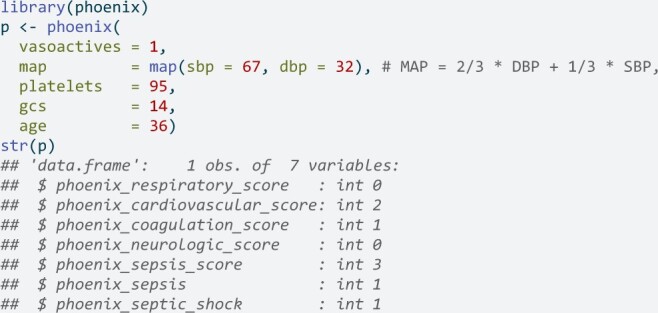


Scoring in Python:


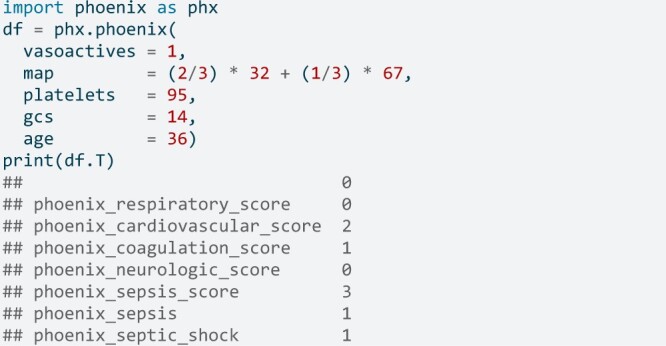


In the above example, only the known data need be inputted. Missing values are mapped to scores of zero. That is, Phoenix is based on the explicitly defined inputs and any missing inputs are implicitly mapping to scores of zero. This is consistent with the Phoenix development process.^4^

To apply the Phoenix rubric to a full dataset in R:


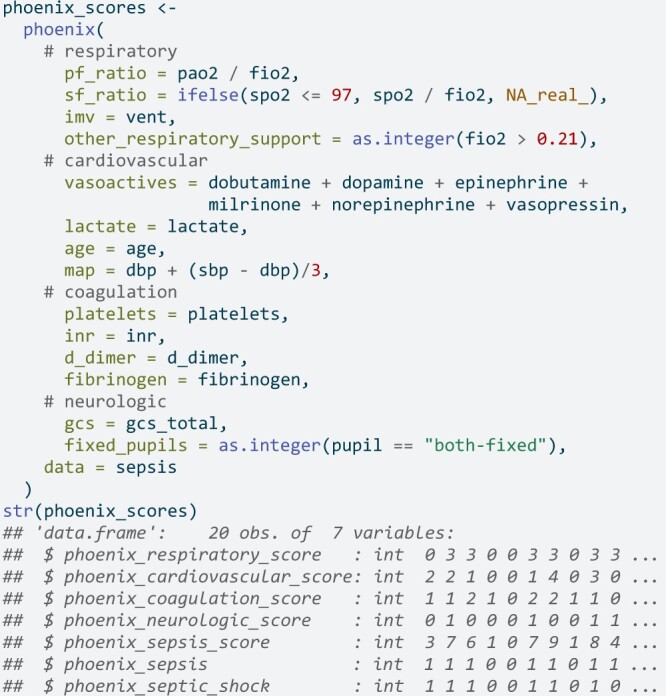


Details on the expected units for inputs as denoted in Table 1 are also provided in the documentation for the R package.


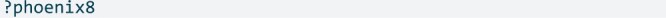


and Python module


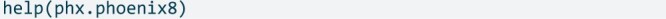


Additional details and examples for each of the 8 organ dysfunction scoring functions, phoenix(), and phoenix8() methods are in the Supplementary Examples. The supplement includes examples in R, Python, and SQLite.

## Discussion

The transition to the Phoenix criteria marks a major change in the conceptual definition of pediatric sepsis.^7^ Applicable across differentially resourced settings, the Phoenix criteria should help improve clinical care and research across the globe.

Additionally, the eponymic R package and Python modules provide researchers a simple to use tool for consistent and faithful application of the Phoenix criteria to any applicable dataset.

Researchers are encouraged to carefully review the provided documentation for the package. Some assumptions used by the package are easy to overlook. The example for using phoenix() on a data.frame in R has expressions for the PaO_2_: FiO_2_ ratio, SpO_2_: FiO_2_ ratio, respiratory support, vasoactive medications, mean arterial pressure (MAP), and fixed pupils. This example is provided to be explicit about data assumptions such as the SpO_2_: FiO_2_ ratio only being valid for SpO_2_ values not exceeding 97.

In practice, we suggest processing the data first such that only a variable name need be passed as an argument. This could be particularly useful in the case of MAP where a hierarchy of values could be used, that is, invasive MAP readings are preferable to calculated MAP based on invasive SBP and DBP, and invasive measurements are preferable to non-invasive blood pressure cuff measurements.

## Conclusion

The phoenix R package and Python module meet the objectives of the FAIR Principles for Research Software (FAIR4RS Principles).^8^ The package and module are intuitive tools for consistently and accurately applying the Phoenix pediatric sepsis criteria to clinical datasets.

## Supplementary Material

ooae066_Supplementary_Data

## Data Availability

The phoenix R package is freely available on CRAN at https://cran.r-project.org/package=phoenix. The phoenix Python module is freely available from PyPi: https://pypi.org/project/phoenix-sepsis/. Extensive documentation and example SQLite queries are available online at https://cu-dbmi-peds.github.io/phoenix/index.html. This manuscript was written using Quarto version 1.4.553 (https://quarto.org/) and R version 4.4.0 (2024-04-24). All R code, materials, and dependencies can be found at https://github.com/cu-dbmi-peds/phoenix_application_note/.
