# Supplementary material for: phoenix: an R package and Python module for calculating the Phoenix pediatric sepsis score and criteria
Source: JAMIA Open. 2024 Jul 4;7(3):ooae066. doi: 10.1093/jamiaopen/ooae066 (PMC11223841; doi:10.1093/jamiaopen/ooae066)
Supplement: ooae066_Supplementary_Data [file ooae066_supplementary_data.pdf]

Supplement Material

phoenix: R package and Python module for calculating the Phoenix Pediatric Sepsis Score and Criteria

Table of contents

1 Installing Software **4**
1.1 R 4
1.2 Python 4

2 Example Data **4**
2.1 R 5
2.2 Python 6
2.3 SQL 8

3 Organ Dysfunction Scores **8**
3.1 Respiratory 10
3.1.1 Inputs: 10
3.1.2 Phoenix Scoring 10
3.1.3 R 11
3.1.4 Python 12
3.1.5 SQLite 12
3.2 Cardiovascular 13
3.2.1 Inputs 13
3.2.2 Phoenix Scoring 14
3.2.3 R 15
3.2.4 Python 16
3.2.5 SQLite 16
3.3 Coagulation 18
3.3.1 Inputs: 18
3.3.2 Phoenix Scoring 18
3.3.3 R 19
3.3.4 Python 19
3.3.5 SQLite 20

3.4 Neurologic 21
3.4.1 Inputs: 21
3.4.2 Phoenix Scoring 21
3.4.3 R 21
3.4.4 Python 22
3.4.5 SQLite 22
3.5 Endocrine 23
3.5.1 Inputs: 23
3.5.2 Phoenix-8 Scoring 23
3.5.3 R 23
3.5.4 Python 23
3.5.5 SQLite 24
3.6 Immunologic 24
3.6.1 Inputs: 24
3.6.2 Phoenix-8 Scoring 25
3.6.3 R 25
3.6.4 Python 25
3.6.5 SQLite 26
3.7 Renal 26
3.7.1 Inputs: 26
3.7.2 Phoenix-8 Scoring 27
3.7.3 R 27
3.7.4 Python 28
3.7.5 SQLite 28
3.8 Hepatic 29
3.8.1 Inputs: 29
3.8.2 Phoenix-8 scoring 29
3.8.3 R 29
3.8.4 Python 30
3.8.5 SQLite 30

4 Phoenix Criteria **31**
4.1 R 31
4.2 Python 32
4.3 SQLite 33

5 Phoenix-8 **34**
5.1 R 34
5.2 Python 36
5.3 SQLite 37

6 Clinical Vignettes **39**
6.1 Clinical Vignette 1 39

6.2 Clinical Vignette 2 40

References **42**

Methods for applying the Phoenix organ dysfunction scoring and criteria in R and Python have been packaged and published. Example SQL queries (SQLite) are also presented here.

1 Installing Software

1.1 R

To install the R package

1. Install R
2. Within R call `install.packages('phoenix')` to get the current released version.

Install the development version of `phoenix` directly from GitHub via the `remotes` package[1]:

```r
if (!("remotes" %in% rownames(installed.packages()))) {
  warning("installing remotes from https://cran.rstudio.com")
  install.packages("remotes", repo = "https://cran.rstudio.com")
}

remotes::install_github("cu-dbmi-peds/phoenix")
```

NOTE: If you are working on a Windows machine, you will need to download and install `Rtools`.

1.2 Python

To install the Python module use `pip`.

```
pip install phoenix-sepsis
```

2 Example Data

A small example data set has been provided in the phoenix R package and the phoenix-sepsis. In R the data set, `sepsis`, is a provided as a data.frame. In Python, it is provided as a .csv file with instructions for loading it into a pandas DataFrame. For the SQL examples we'll use a in-memory SQLite database and load the data into a table called `sepsis`.

The data set consists of 20 observations of 27 variables. The column names and ordering are consistent between the R package and Python module.

Column Name	Description
pid	patient identification number
age	age in months
fio2	fraction of inspired oxygen
pao2	partial pressure of oxygen in arterial blood (mmHg)
spo2	pulse oximetry
vent	indicator for invasive mechanical ventilation
gcs_total	total Glasgow Coma Scale
pupil	character vector reporting if pupils are reactive or fixed.
platelets	platelets measured in 1,000 / μL
inr	international normalized ratio
d_dimer	D-dimer; units of mg/L FEU
fibrinogen	units of mg/dL
dbp	diagnostic blood pressure (mmHg)
sbp	systolic blood pressure (mmHg)
lactate	units of mmol/L
dobutamine	indicator for receiving systemic dobutamine
dopamine	indicator for receiving systemic dopamine
epinephrine	indicator for receiving systemic epinephrine
milrinone	indicator for receiving systemic milrinone
norepinephrine	indicator for receiving systemic norepinephrine
vasopressin	indicator for receiving systemic vasopressin
glucose	units of mg/dL
anc	units of 1,000 cells per cubic millimeter
alc	units of 1,000 cells per cubic millimeter
creatinine	units of mg/dL
bilirubin	units of mg/dL
alt	units of IU/L

2.1 R

The `sepsis` data set is lazyloaded when the package namespace is loaded and attached. No other packages are needed to run the R examples in this supplement.

```
library(phoenix)
str(sepsis)
## 'data.frame':    20 obs. of  27 variables:
##  $ pid           : int  1 2 3 4 5 6 7 8 9 10 ...
##  $ age           : num  0.06 201.7 20.8 192.5 214.4 ...
##  $ fio2          : num  0.75 0.75 1 NA NA 0.6 0.5 0.3 0.65 0.8 ...
##  $ pao2          : num  NA 75.3 49.5 NA 38.7 69.9 NA NA 51 NA ...
```

```
## $ spo2          : int  99 95 NA NA 95 88 31 97 82 76 ...
## $ vent          : int  1 1 1 0 0 1 1 1 1 1 ...
## $ gcs_total     : int  NA 5 15 14 NA 3 NA 15 3 3 ...
## $ pupil         : chr  "" "both-reactive" "both-reactive" "" ...
## $ platelets     : int  199 243 49 NA 393 86 65 215 101 292 ...
## $ inr           : num  1.46 1.18 1.6 1.3 NA 1.23 3.1 0.97 1.08 NA ...
## $ d_dimer       : num  NA 2.45 NA 2.82 NA 4.72 NA 5.15 7.71 NA ...
## $ fibrinogen    : int  180 311 309 220 NA 270 94 489 456 NA ...
## $ dbp           : int  40 60 87 57 57 79 11 66 51 58 ...
## $ sbp           : int  53 90 233 104 101 119 14 112 117 84 ...
## $ lactate       : num  NA 3.32 1 NA NA 1.15 NA NA 8.1 NA ...
## $ dobutamine    : int  1 0 0 0 0 0 0 0 0 0 ...
## $ dopamine      : int  1 1 1 0 0 1 0 0 0 0 ...
## $ epinephrine   : int  1 0 0 0 0 0 1 0 1 0 ...
## $ milrinone     : int  1 0 0 0 0 0 1 0 1 0 ...
## $ norepinephrine: int  0 1 0 0 0 0 0 0 1 0 ...
## $ vasopressin   : int  0 0 0 0 0 0 1 0 1 0 ...
## $ glucose       : num  NA 110 93 110 NA 147 NA 100 264 93 ...
## $ anc           : num  NA 14.22 2.21 3.18 NA ...
## $ alc           : num  NA 2.22 0.19 0.645 NA ...
## $ creatinine    : num  1.03 0.51 0.33 0.31 0.52 0.77 1.47 0.58 1.23 0.18 ...
## $ bilirubin     : num  NA 0.2 0.8 8.5 NA 1.2 1.7 0.5 21.1 NA ...
## $ alt           : int  36 32 182 21 NA 15 3664 50 151 NA ...
```

2.2 Python

Along with with `phoenix` module, you will need `numpy`, `pandas`, and `importlib.resources` to run all the Python examples in this supplement.

```
import numpy as np
import pandas as pd
import importlib.resources
import phoenix as phx

path = importlib.resources.files('phoenix')
sepsis = pd.read_csv(path.joinpath('data').joinpath('sepsis.csv'))

print(sepsis.info())
## <class 'pandas.core.frame.DataFrame'>
## RangeIndex: 20 entries, 0 to 19
## Data columns (total 27 columns):
```

```
##  #   Column          Non-Null Count  Dtype
## ---  ------          --------------  -----
##  0   pid             20 non-null     int64
##  1   age             20 non-null     float64
##  2   fio2            16 non-null     float64
##  3   pao2            9 non-null      float64
##  4   spo2            15 non-null     float64
##  5   vent            20 non-null     int64
##  6   gcs_total       9 non-null      float64
##  7   pupil           8 non-null      object
##  8   platelets       16 non-null     float64
##  9   inr             15 non-null     float64
##  10  d_dimer         8 non-null      float64
##  11  fibrinogen      13 non-null     float64
##  12  dbp             20 non-null     int64
##  13  sbp             20 non-null     int64
##  14  lactate         7 non-null      float64
##  15  dobutamine      20 non-null     int64
##  16  dopamine        20 non-null     int64
##  17  epinephrine     20 non-null     int64
##  18  milrinone       20 non-null     int64
##  19  norepinephrine  20 non-null     int64
##  20  vasopressin     20 non-null     int64
##  21  glucose         12 non-null     float64
##  22  anc             11 non-null     float64
##  23  alc             11 non-null     float64
##  24  creatinine      18 non-null     float64
##  25  bilirubin       14 non-null     float64
##  26  alt             14 non-null     float64
## dtypes: float64(16), int64(10), object(1)
## memory usage: 4.3+ KB
## None
print(sepsis.head())
##    pid     age  fio2  pao2  spo2  ...     anc    alc creatinine  bilirubin    alt
## 0    1    0.06  0.75   NaN  99.0  ...     NaN    NaN       1.03        NaN   36.0
## 1    2  201.70  0.75  75.3  95.0  ...  14.220  2.220       0.51        0.2   32.0
## 2    3   20.80  1.00  49.5   NaN  ...   2.210  0.190       0.33        0.8  182.0
## 3    4  192.50   NaN   NaN   NaN  ...   3.184  0.645       0.31        8.5   21.0
## 4    5  214.40   NaN  38.7  95.0  ...     NaN    NaN       0.52        NaN    NaN
##
## [5 rows x 27 columns]
```

2.3 SQL

The example SQL queries are done in a in-memory SQLite instance set up within R. The following code chunk sets up the database with the example sepsis data loaded into a table of the same name.

```
library(odbc)
library(DBI)
library(RSQLite)
library(phoenix)
con <- dbConnect(drv = RSQLite::SQLite(), dbname = ":memory:")
dbWriteTable(conn = con, name = "sepsis", value = sepsis)
```

The structure of the table:

```
PRAGMA table_info(sepsis);
```

Table 2: Displaying records 1 - 10

cid	name	type	notnull	dflt_value	pk
0	pid	INTEGER	0	NA	0
1	age	REAL	0	NA	0
2	fio2	REAL	0	NA	0
3	pao2	REAL	0	NA	0
4	spo2	INTEGER	0	NA	0
5	vent	INTEGER	0	NA	0
6	gcs_total	INTEGER	0	NA	0
7	pupil	TEXT	0	NA	0
8	platelets	INTEGER	0	NA	0
9	inr	REAL	0	NA	0

3 Organ Dysfunction Scores

In each of the following eight sections we present details on the Phoenix scoring and provide examples of applying the Phoenix scoring in R, Python, and SQL.

Important Notes:

- Phoenix was developed on a data set of pediatric (age 0 to 18 years) non-birth hospitalizations.

- Phoenix was developed assuming that missing data maps to a score of zero. In the source code for the R package and Python modules you can see that missing values are mapped to “healthy” values such that a score of 0 will be returned. The example SQL queries do this explicitly.

  This means any omitted arguments will map to scores of zero where the omitted variable is needed. Thus, the organ dysfunction scores, and the composite scores are based on the explicitly used inputs.

- The provided example data set provides all the needed inputs for the Phoenix criteria, but requires some processing before scoring. For example, the SpO_2:FiO_2 ratio that is needed for the respiratory score is not provided in the `sepsis` data set. The componets, `spo2` and `fio2`, are part of the data set. The reason for this is to highlight an important restriction on the use of the SpO_2:FiO_2 ratio: it is only valid for SpO_2 values least than or equal to 97.

  Processing the data and creating specific columns for the needed inputs, as seen below, would be preferable in practice, for example,

```
sepsis2 <-
  with(sepsis,
    data.frame(
        pfr = pao2 / fio2,
        sfr = ifelse(spo2 <= 97, spo2 / fio2, NA_real_),
        imv = vent,
        ors = as.integer(fio2 > 0.21),
        vasos = dobutamine + dopamine + epinephrine + milrinone + norepinephrine + vasopress
        lactate = lactate,
        age = age,
        map = map(sbp = sbp, dbp = dbp),
        plts = platelets,
        inr = inr,
        ddimer = d_dimer,
        fib = fibrinogen,
        gcs = gcs_total,
        pupils = as.integer(pupil == "both-fixed"),
        glucose = glucose,
        anc = anc,
        alc = alc,
        creatinine = creatinine,
        bilirubin = bilirubin,
        alt = alt)
  )
```

The examples that follow will use the `sepsis` data set to highlight the data assumptions.

3.1 Respiratory

The Phoenix scoring for respiratory dysfunction is based on the pSOFA [2] criteria.

3.1.1 Inputs:

- `pf_ratio`: is the ratio of PaO_2 (partial pressure or oxygen in arterial blood, units of mmHg) to FiO_2 (fraction of inspiratory oxygen, values expected to be between 0.21 for room air, to 1.00). Gathering the PaO_2 is an invasive procedure.
- `sf_ratio`: The SpO_2 (pulse oximetry) to FiO_2 ratio is a non-invasive surrogate for the PF ratio. Important note: during the development of the Phoenix criteria SF ratios were only valid to consider if the SpO_2 is ≤ 97. Expected value range for SpO_2 is 0 to 100.
- `imv`: Invasive mechanical ventilation. This is an integer valued indicator variable: 0 = not intubated; 1 = intubated.
- `other_respiratory_support`: Any oxygen support, e.g., high-flow, non-invasive positive pressure, or IMV. This can be inferred if FiO_2 exceeds 0.21.

3.1.2 Phoenix Scoring

The respiratory score is based on the PaO_2:FiO_2 ratio or the SpO_2:FiO_2 ratio. PaO_2:FiO_2 is used preferentially over the SpO_2:FiO_2 with the worst possible score used. That is, if a patient would have 1 point based on PaO_2:FiO_2 but 2 points based on SpO_2:FiO_2, then the score is 2 points.

	0 Points	1 Point	2 Points	3 Points
		Any respiratory support	IMV	IMV
PaO_2:FiO_2	≥ 400	< 400	< 200	< 100
SpO_2:FiO_2	≥ 292	< 292	< 220	< 148

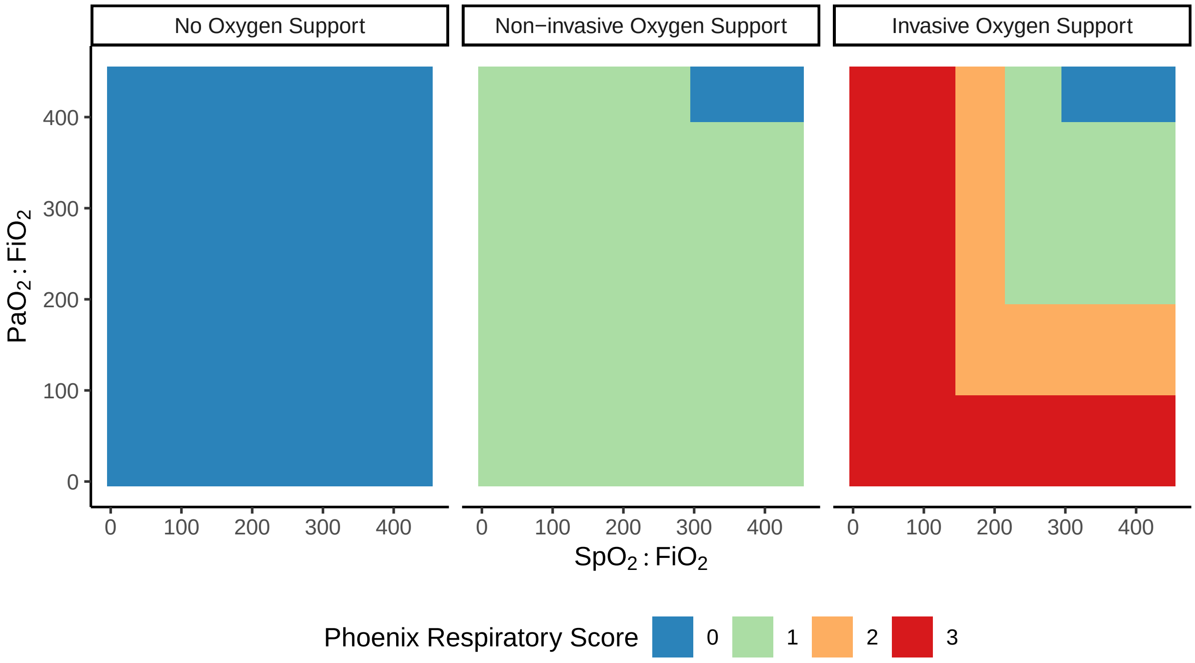

3.1.3 R

The `phoenix_respiratory` call in R returns an integer vector of scores. The inputs are expected to be vectors of equal length and can be explicitly defined from a data set or short handed by passing the data set in via the `data` argument as shown below.

```
phoenix_respiratory(
  pf_ratio = pao2 / fio2,
  sf_ratio = ifelse(spo2 <= 97, spo2 / fio2, NA_real_),
  imv = vent,
  other_respiratory_support = as.integer(fio2 > 0.21),
  data = sepsis
)
##  [1] 0 3 3 0 0 3 3 0 3 3 3 1 0 2 3 0 2 3 2 0
```

The above explicitly shows the data assumptions for the SpO_2:FiO_2 ratio (`sf_ratio`) and `other_respiratory_support`. If the data has be processed with these assumptions already, or if you only have the SpO_2:FiO_2 ratio, the inputs can be simplified as

```
phoenix_respiratory(
  pf_ratio = pfr,
  sf_ratio = sfr,
```

```
  imv = imv,
  other_respiratory_support = ors,
  data = sepsis2
)
##  [1] 0 3 3 0 0 3 3 0 3 3 3 1 0 2 3 0 2 3 2 0
```

3.1.4 Python

The Python implementation is similar to the implementation in R. The notable difference in the lack of the `data` argument.

```
py_resp = phx.phoenix_respiratory(
    pf_ratio = sepsis["pao2"] / sepsis["fio2"],
    sf_ratio = np.where(sepsis["spo2"] <= 97, sepsis["spo2"] / sepsis["fio2"], np.nan),
    imv      = sepsis["vent"],
    other_respiratory_support = (sepsis["fio2"] > 0.21).astype(int).to_numpy()
)
print(type(py_resp))
## <class 'numpy.ndarray'>
print(py_resp)
## [0 3 3 0 0 3 3 0 3 3 3 1 0 2 3 0 2 3 2 0]
```

3.1.5 SQLite

For the SQLite example we start by constructing the needed variables form the `sepsis` table and then building the score. The results will be stored in new table so we can use them again when assessing the Phoenix and Phoenix-8 scores.

```
CREATE TABLE IF NOT EXISTS respiratory AS
  SELECT
    pid,
    (
      imv * (
        IIF(pfr < 100 OR sfr < 148, 1, 0) + IIF(pfr < 200 OR sfr < 220, 1, 0)
      ) +
      other_respiratory_support * IIF(pfr < 400 OR sfr < 292, 1, 0)
    ) AS phoenix_respiratory_score
  FROM
  (
    SELECT
```

```
    pid,
    COALESCE(pao2 / fio2, 500) AS pfr,
    COALESCE(IIF(spo2 <= 97, spo2 / fio2, 500), 500) AS sfr,
    COALESCE(vent, 0) AS imv,
    IIF(fio2 > 0.21 OR vent = 1, 1, 0) AS other_respiratory_support
  FROM sepsis
);
```

```
SELECT * from respiratory
```

Table 4: Displaying records 1 - 10

pid	phoenix_respiratory_score
1	0
2	3
3	3
4	0
5	0
6	3
7	3
8	0
9	3
10	3

3.2 Cardiovascular

There are three components of the Phoenix scoring for cardiovascular dysfunction. The number of systemic vasoactive medications is a modification of the vasoactive-inotropic score[3] (VIS) and the cardiovascular score from the Pediatric Logistic Organ Dysfunction-2 (PELOD-2)[4].

3.2.1 Inputs

- `vasoactives`: is an integer count of the number of systemic vasoactive medications the patient is currently receiving. During development of the Phoenix criteria it was found that just the count of the medications was sufficient to be useful, the dosage was not needed. There were six medications considered: dobutamine, dopamine, epinephrine, milrinone, norepinephrine, and vasopressin. Again, it is systemic use of the medication that is important. For example, an injection of epinephrine to halt an allergic reaction would not count, whereas having an epinephrine drip to treat hypotension or bradycardia would count.

- `lactate`: level of lactate in the blood, measured in mmol/L
- `age`: in months
- `map`: mean arterial pressure (mmHg). During development of the Phoenix criteria, map, and blood pressure values in general, obtained from arterial measures were used preferentially over values obtained from cuffs. Reported values were used preferentially over calculated values. If you need to calculate the map use DBP + (1/3) * (SBP - DBP) where DBP is diastolic blood pressure (mmHg) and SBP is systolic blood pressure (mmHg).

3.2.2 Phoenix Scoring

The Phoenix score is the sum of the vasoactive medications score, lactate score, and mean MAP score for a total score ranging between 0 and 6 points.

	0 Points	1 Point	2 Points
Systemic Vasoactive Medications	No medications	1 medication	2 or more medications
Lactate (mmol/L)	< 5	$5 \leq \text{Lactate} < 11$	≥ 11
Age (months) adjusted MAP (mmHg)			
$0 \leq \text{Age} < 1$	≥ 31	$17 \leq \text{MAP} < 31$	< 17
$1 \leq \text{Age} < 12$	≥ 39	$25 \leq \text{MAP} < 39$	< 25
$12 \leq \text{Age} < 24$	≥ 44	$31 \leq \text{MAP} < 44$	< 31
$24 \leq \text{Age} < 60$	≥ 45	$32 \leq \text{MAP} < 45$	< 32
$60 \leq \text{Age} < 144$	≥ 49	$36 \leq \text{MAP} < 49$	< 36
$144 \leq \text{Age} < 216$	≥ 52	$38 \leq \text{MAP} < 52$	< 38

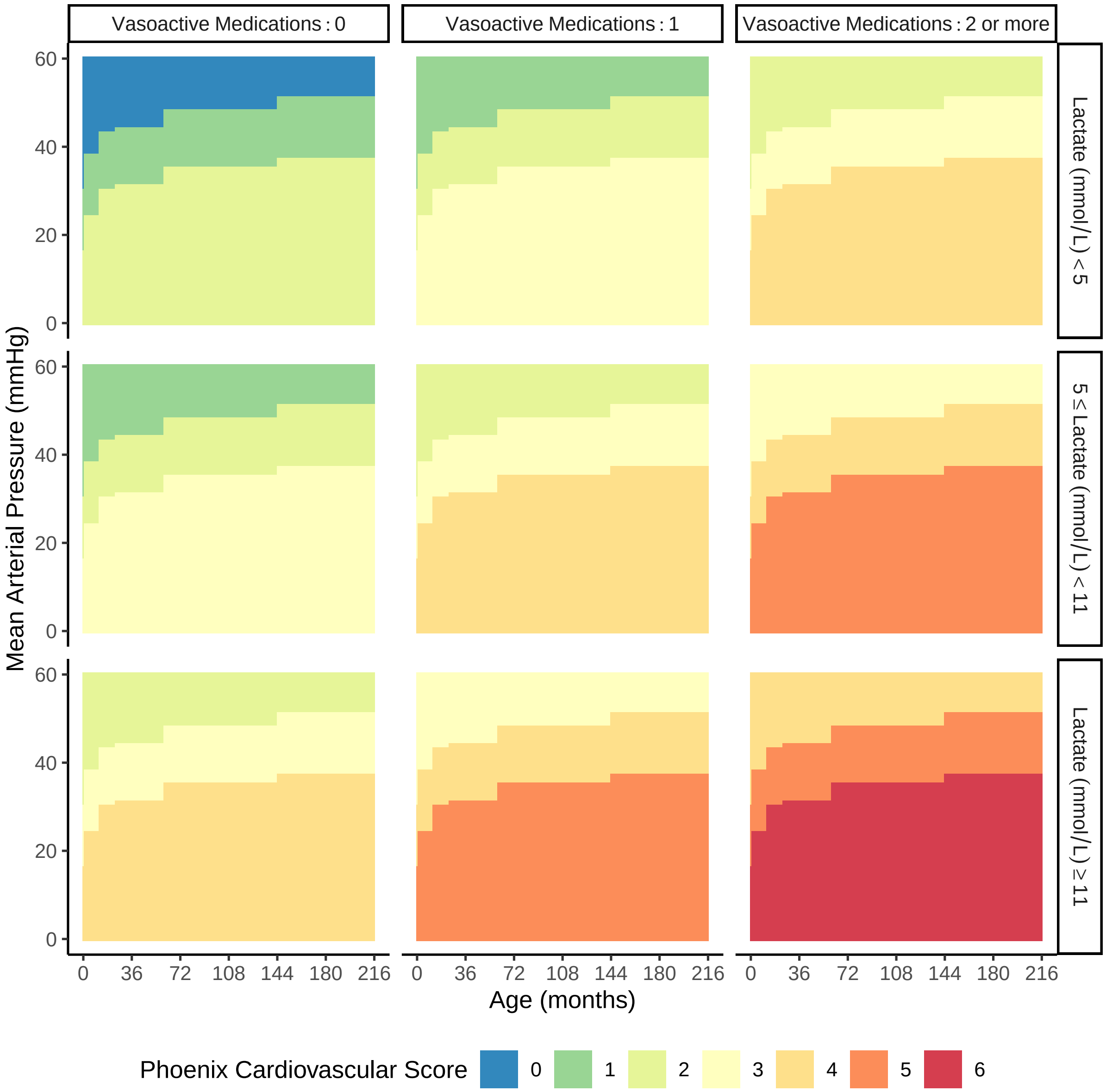

3.2.3 R

For this example data set we can specify a sum for the `vasoactives` since each of the columns are indicators. The mean arterial pressure is estimated based on the reported systolic and diastolic pressures. The R package `phoenix` provides the function `map` to simplify the estimate: $MAP = \frac{2}{3}DBP + \frac{1}{3}SBP$.

```r
phoenix_cardiovascular(
  vasoactives = dobutamine + dopamine + epinephrine +
                milrinone + norepinephrine + vasopressin,
  lactate = lactate,
  age = age,
  map = map(sbp, dbp),
  data = sepsis
)
##  [1] 2 2 1 0 0 1 4 0 3 0 3 0 0 2 3 2 2 2 2 1
```

3.2.4 Python

As with the R package, the Python module provides the function `map` to simplify the estimation of the mean arterial pressure.

```python
py_card = phx.phoenix_cardiovascular(
    vasoactives = sepsis["dobutamine"] + sepsis["dopamine"] + sepsis["epinephrine"] +
                  sepsis["milrinone"] + sepsis["norepinephrine"] + sepsis["vasopressin"],
    lactate = sepsis["lactate"],
    age = sepsis["age"],
    map = phx.map(sepsis["sbp"], sepsis["dbp"])
)
print(type(py_card))
## <class 'numpy.ndarray'>
print(py_card)
## [2 2 1 0 0 1 4 0 3 0 3 0 0 2 3 2 2 2 2 1]
```

3.2.5 SQLite

For the SQLite example we will create a new table to store the results which will be used when getting the overall Phoenix and Phoenix-8 scores.

```sql
CREATE TABLE IF NOT EXISTS cardiovascular AS

SELECT
  pid,
  vaso_points + lactate_points + map_points AS phoenix_cardiovascular_score
FROM
(
  SELECT *,
```

```sql
    CASE WHEN vasos > 1 THEN 2
         WHEN vasos > 0 THEN 1
         ELSE 0 END AS vaso_points,
    CASE WHEN lactate >= 11 THEN 2
         WHEN lactate >=  5 THEN 1
         ELSE 0 END AS lactate_points,
    CASE WHEN (age >=   0 AND age <    1) AND (map < 17) THEN 2
         WHEN (age >=   1 AND age <   12) AND (map < 25) THEN 2
         WHEN (age >=  12 AND age <   24) AND (map < 31) THEN 2
         WHEN (age >=  24 AND age <   60) AND (map < 32) THEN 2
         WHEN (age >=  60 AND age <  144) AND (map < 36) THEN 2
         WHEN (age >= 144 AND age <= 216) AND (map < 38) THEN 2
         WHEN (age >=   0 AND age <    1) AND (map < 31) THEN 1
         WHEN (age >=   1 AND age <   12) AND (map < 39) THEN 1
         WHEN (age >=  12 AND age <   24) AND (map < 44) THEN 1
         WHEN (age >=  24 AND age <   60) AND (map < 45) THEN 1
         WHEN (age >=  60 AND age <  144) AND (map < 49) THEN 1
         WHEN (age >= 144 AND age <= 216) AND (map < 52) THEN 1
         ELSE 0 END AS map_points
  FROM
  (
    SELECT
      pid,
      COALESCE(dobutamine, 0) +
        COALESCE(dopamine, 0) +
        COALESCE(epinephrine, 0) +
        COALESCE(milrinone, 0) +
        COALESCE(norepinephrine, 0) +
        COALESCE(vasopressin, 0) AS vasos,
      lactate,
      dbp + (sbp - dbp) / 3 AS map,
      age
    FROM sepsis
  )
);
```

```sql
SELECT * FROM cardiovascular;
```

Table 6: Displaying records 1 - 10

pid	phoenix_cardiovascular_score
1	2
2	2
3	1
4	0
5	0
6	1
7	4
8	0
9	3
10	0

3.3 Coagulation

The Disseminated intravascular coagulation score (DIC)[5] is the basis for the Phoenix coagulation dysfunction score.

3.3.1 Inputs:

- `platelets`: in units of 1,000 / μL
- `inr`: international normalized ratio; a metric for time require for blood to clot.
- `d_dimer`: in units of mg/L FEU
- `fibrinogen` : in units of mg/dL

3.3.2 Phoenix Scoring

While there are four components to this score, the maximum number of points assigned is 2.

	0 Points	1 Point
Platelets (1000/muL)	≥ 100	< 100
INR	≤ 1.3	> 1.3
D-Dimer (mg/L FEU)	≤ 2	> 2
Fibrinogen (mg/dL)	≥ 100	< 100

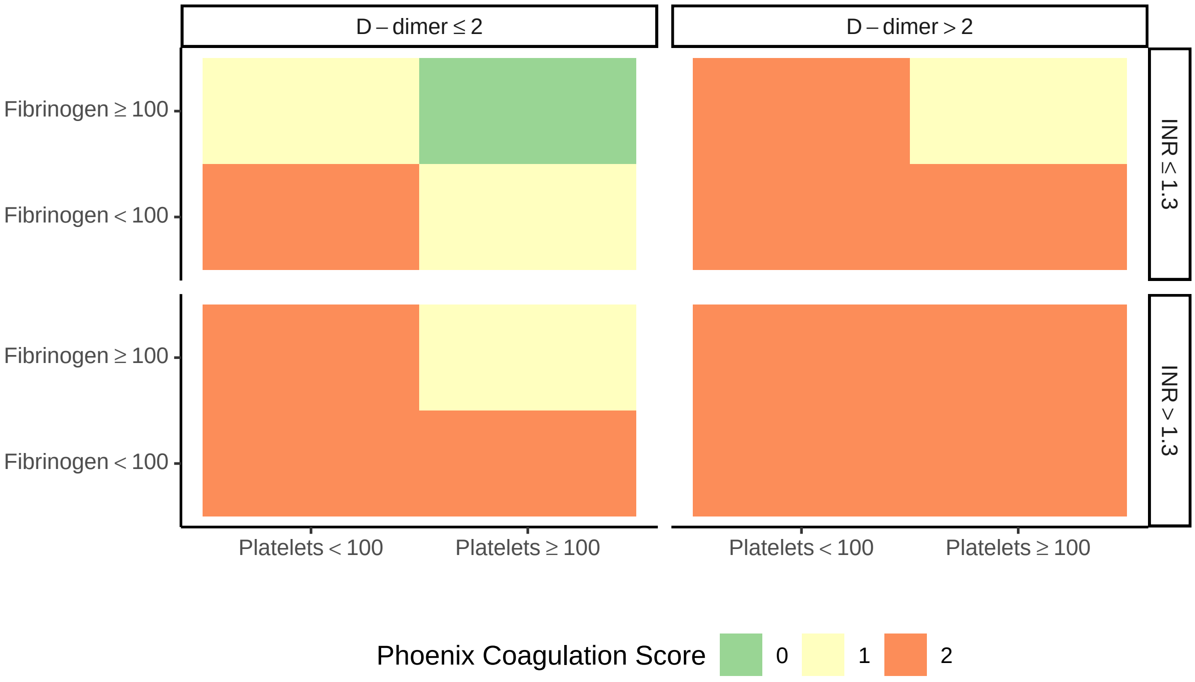

3.3.3 R

The needed inputs for the coagulation score are as is within the example data set.

```
phoenix_coagulation(
  platelets = platelets,
  inr = inr,
  d_dimer = d_dimer,
  fibrinogen = fibrinogen,
  data = sepsis
)
##  [1] 1 1 2 1 0 2 2 1 1 0 1 0 0 1 2 1 1 2 0 1
```

3.3.4 Python

The needed inputs for the coagulation score are as is within the example data set.

```
py_coag = phx.phoenix_coagulation(
    platelets = sepsis['platelets'],
    inr = sepsis['inr'],
    d_dimer = sepsis['d_dimer'],
```

```
    fibrinogen = sepsis['fibrinogen']
)
print(type(py_coag))
## <class 'numpy.ndarray'>
print(py_coag)
## [1 1 2 1 0 2 2 1 1 0 1 0 0 1 2 1 1 2 0 1]
```

3.3.5 SQLite

We will create a coagulation table to use when assessing the Phoenix and Phoenix-8 scores.

```
CREATE TABLE IF NOT EXISTS coagulation AS

SELECT
  pid,
  CASE WHEN plts + inr + ddm + fib >= 2 THEN 2
       ELSE plts + inr + ddm + fib END AS phoenix_coagulation_score
FROM (
  SELECT
    pid,
    CASE WHEN platelets < 100  THEN 1 ELSE 0 END AS plts,
    CASE WHEN inr > 1.3        THEN 1 ELSE 0 END AS inr,
    CASE WHEN d_dimer > 2      THEN 1 ELSE 0 END AS ddm,
    CASE WHEN fibrinogen < 100 THEN 1 ELSE 0 END AS fib
  FROM sepsis
)
```

```
SELECT * FROM coagulation
```

Table 8: Displaying records 1 - 10

pid	phoenix_coagulation_score
1	1
2	1
3	2
4	1
5	0
6	2
7	2
8	1

pid	phoenix_coagulation_score
9	1
10	0

3.4 Neurologic

The Phoenix neurologic dysfunction scoring is based on the PELOD-2 [4] neurologic dysfunction scoring.

3.4.1 Inputs:

- `gcs`: an integer vector for the (total) Glasgow Comma Score. The total score is the sum of the eye, verbal, and motor scores. Valid gcs values are integers 3, 4, 5, ..., 14, 15.
- `fixed_pupils`: an integer vector of zeros and ones. 1 = bilaterally fixed pupils, 0 otherwise.

3.4.2 Phoenix Scoring

	0 Points	1 Point	2 Points
	GCS ≥ 11	GCS ≤ 10	Bilaterally fixed pupils

3.4.3 R

The example data reports pupil reactivity as either "both-fixed", "both-reactive", or " ". We need only create an indicator on the fly to build the neurologic score.

```
phoenix_neurologic(
  gcs = gcs_total,
  fixed_pupils = as.integer(pupil == "both-fixed"),
  data = sepsis
)
##  [1] 0 1 0 0 0 1 0 0 1 1 2 0 0 0 0 0 0 0 0 0
```

3.4.4 Python

```
py_neur = phx.phoenix_neurologic(
    gcs = sepsis["gcs_total"],
    fixed_pupils = (sepsis["pupil"] == "both-fixed").astype(int)
)
print(type(py_neur))
## <class 'numpy.ndarray'>
print(py_neur)
## [0 1 0 0 0 1 0 0 1 1 2 0 0 0 0 0 0 0 0 0]
```

3.4.5 SQLite

```
CREATE TABLE IF NOT EXISTS neurologic AS

SELECT
  pid,
  CASE WHEN fixed_pupils = 1 THEN 2
       WHEN gcs = 1          THEN 1
       ELSE 0 END AS phoenix_neurologic_score
FROM (
  SELECT
    pid,
    CASE WHEN gcs_total <= 10 THEN 1 ELSE 0 END AS gcs,
    CASE WHEN pupil = "both-fixed" THEN 1 ELSE 0 END AS fixed_pupils
  FROM sepsis
);
```

```
SELECT * FROM neurologic;
```

Table 10: Displaying records 1 - 10

pid	phoenix_neurologic_score
1	0
2	1
3	0
4	0
5	0

pid	phoenix_neurologic_score
6	1
7	0
8	0
9	1
10	1

3.5 Endocrine

Endocrine dysfunction is only part of the Phoenix-8 scoring and is based on a subset of the Pediatric organ dysfunction information update mandate (PODIUM)[6] endocrine thresholds.

3.5.1 Inputs:

- `glucose`: blood glucose in units of mg/dL

3.5.2 Phoenix-8 Scoring

	0 Points	1 Point
Blood Glucose (mg/dL)	$50 \leq$ Blood Glucose ≤ 150	< 50; or > 150

3.5.3 R

```
phoenix_endocrine(glucose = glucose, data = sepsis)
##  [1] 0 0 0 0 0 0 0 0 1 0 1 0 0 0 0 0 0 0 0 1
```

3.5.4 Python

```
py_endo = phx.phoenix_endocrine(sepsis["glucose"])
print(type(py_endo))
## <class 'numpy.ndarray'>
print(py_endo)
## [0 0 0 0 0 0 0 0 1 0 1 0 0 0 0 0 0 0 0 1]
```

3.5.5 SQLite

```
CREATE TABLE IF NOT EXISTS endocrine AS
SELECT
  pid,
  CASE WHEN glucose <  50 THEN 1
       WHEN glucose > 150 THEN 1
       ELSE 0 END AS phoenix_endocrine_score
FROM sepsis
```

```
SELECT * FROM endocrine
```

Table 12: Displaying records 1 - 10

pid	phoenix_endocrine_score
1	0
2	0
3	0
4	0
5	0
6	0
7	0
8	0
9	1
10	0

3.6 Immunologic

Immunologic dysfunction is only part of the Phoenix-8 scoring and is based on a subset of the Pediatric organ dysfunction information update mandate (PODIUM)[6] immunologic thresholds.

3.6.1 Inputs:

- `anc`: Absolute neutrophil count in units of 1,000 cells per cubic millimeter
- `alc`: absolute lymphocyte count in units of 1,000 cells per cubic millimeter

3.6.2 Phoenix-8 Scoring

	0 Points	1 Point
ANC (cells/mm^3)	≥ 500	< 500
ALC (cells/mm^3)	≥ 1000	< 1000

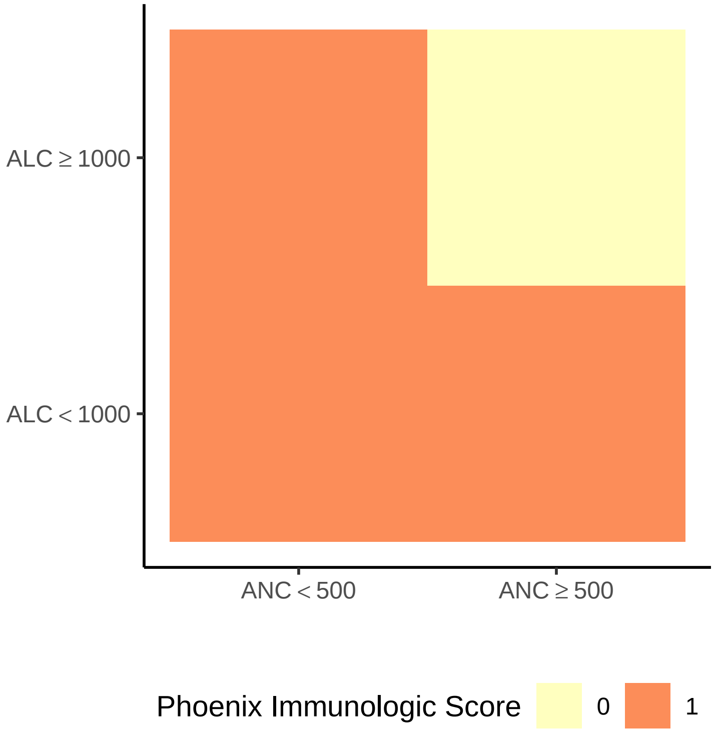

3.6.3 R

```
phoenix_immunologic(anc = anc, alc = alc, data = sepsis)
##  [1] 0 1 1 1 0 1 0 1 1 1 0 0 0 0 0 1 1 0 1 1
```

3.6.4 Python

```
py_immu = phx.phoenix_immunologic(sepsis["anc"], sepsis["alc"])
print(type(py_immu))
## <class 'numpy.ndarray'>
```

```
print(py_immu)
## [0 1 1 1 0 1 0 1 1 1 0 0 0 0 0 1 1 0 1 1]
```

3.6.5 SQLite

```
CREATE TABLE IF NOT EXISTS immunologic AS
SELECT
  pid,
  CASE WHEN anc <  500 THEN 1
       WHEN alc < 1000 THEN 1
       ELSE 0 END AS phoenix_immunologic_score
FROM sepsis
```

```
SELECT * FROM immunologic
```

Table 14: Displaying records 1 - 10

pid	phoenix_immunologic_score
1	0
2	1
3	1
4	1
5	0
6	1
7	0
8	1
9	1
10	1

3.7 Renal

The renal dysfunction score is only part of the Phoenix-8 scoring and comes from pSOFA[2].

3.7.1 Inputs:

- `creatinine` in units of mg/dL
- `age` in months

3.7.2 Phoenix-8 Scoring

	0 Points	1 Point
Age (months) adjusted Creatinine (mg/dL)		
$0 \leq \text{Age} < 1$	< 0.8	≥ 0.8
$1 \leq \text{Age} < 12$	< 0.3	≥ 0.3
$12 \leq \text{Age} < 24$	< 0.4	≥ 0.4
$24 \leq \text{Age} < 60$	< 0.6	≥ 0.6
$60 \leq \text{Age} < 144$	< 0.7	≥ 0.7
$144 \leq \text{Age} < 216$	< 1.0	≥ 1.0

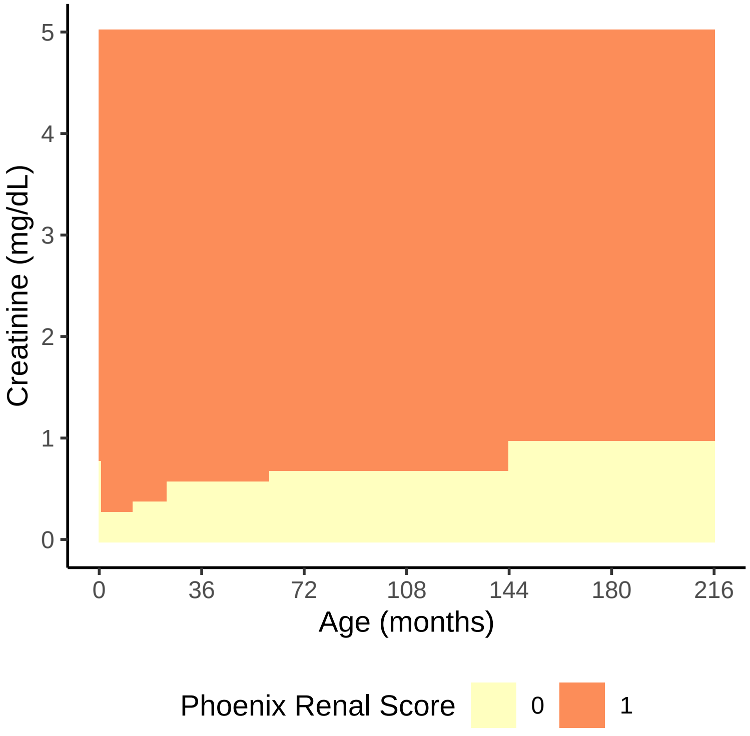

3.7.3 R

```
phoenix_renal(creatinine = creatinine, age = age, data = sepsis)
##  [1] 1 0 0 0 0 1 1 0 1 0 1 0 0 0 1 0 0 1 1 0
```

3.7.4 Python

```
py_renal = phx.phoenix_renal(sepsis["creatinine"], sepsis["age"])
print(type(py_renal))
## <class 'numpy.ndarray'>
print(py_renal)
## [1 0 0 0 0 1 1 0 1 0 1 0 0 0 1 0 0 1 1 0]
```

3.7.5 SQLite

```
CREATE TABLE IF NOT EXISTS renal AS
SELECT
  pid,
  CASE WHEN (age >=   0 AND age <    1) AND (creatinine >= 0.8) THEN 1
       WHEN (age >=   1 AND age <   12) AND (creatinine >= 0.3) THEN 1
       WHEN (age >=  12 AND age <   24) AND (creatinine >= 0.4) THEN 1
       WHEN (age >=  24 AND age <   60) AND (creatinine >= 0.6) THEN 1
       WHEN (age >=  60 AND age <  144) AND (creatinine >= 0.7) THEN 1
       WHEN (age >= 144 AND age <= 216) AND (creatinine >= 1.0) THEN 1
       ELSE 0 END AS phoenix_renal_score
FROM sepsis
```

```
SELECT * FROM renal
```

Table 16: Displaying records 1 - 10

pid	phoenix_renal_score
1	1
2	0
3	0
4	0
5	0
6	1
7	1
8	0
9	1
10	0

3.8 Hepatic

The hepatic scoring is only part of the Phoenix-8 score and comes from the IPSCC[7] criteria.

3.8.1 Inputs:

- `bilirubin`: total bilirubin in units of mg/dL
- `alt`: alanine aminotransferase in units of mg/dL

3.8.2 Phoenix-8 scoring

	0 Points	1 Point
Total Bilirubin (mg/dL)	< 4	≥ 4
ALT (IU/L)	≤ 102	> 102

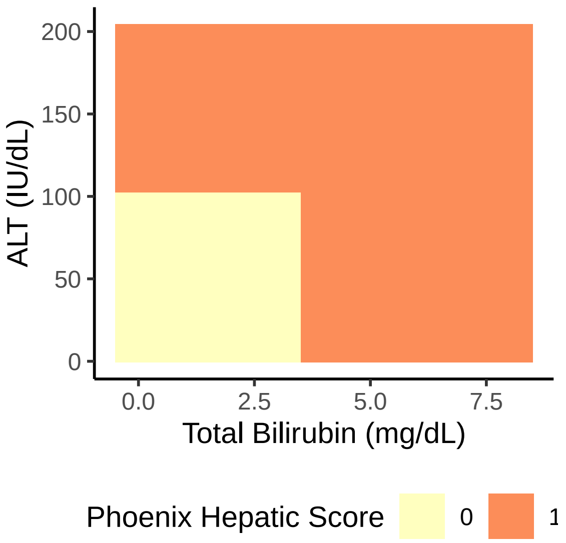

3.8.3 R

Calling `phoenix_hepatic` will return an integer vector of scores.

```
phoenix_hepatic(bilirubin = bilirubin, alt = alt, data = sepsis)
##  [1] 0 0 1 1 0 0 1 0 1 0 0 0 0 0 1 0 0 1 0 0
```

3.8.4 Python

Calling `phoenix_hepatic` will return an integer valued numpy array.

```
py_hepatic = phx.phoenix_hepatic(sepsis["bilirubin"], sepsis["alt"])
print(type(py_hepatic))
## <class 'numpy.ndarray'>
print(py_hepatic)
## [0 0 1 1 0 0 1 0 1 0 0 0 0 0 1 0 0 1 0 0]
```

3.8.5 SQLite

```
CREATE TABLE IF NOT EXISTS hepatic AS

  SELECT
    pid,
    CASE WHEN bilirubin >= 4 THEN 1
         WHEN alt > 102 THEN 1
         ELSE 0 END AS phoenix_hepatic_score
  FROM sepsis
```

```
SELECT * FROM hepatic;
```

Table 18: Displaying records 1 - 10

pid	phoenix_hepatic_score
1	0
2	0
3	1
4	1
5	0
6	0
7	1
8	0
9	1
10	0

4 Phoenix Criteria

The Phoenix Criteria for sepsis is:

- Suspected (or confirmed) infection: define in development as at least one dose of a systemic antimicrobial medication and at least one test for an infection ordered. Development of the criteria restricted these actions to the first 24 hours of an hospital encounter to minimize potential impacts from hospital acquired infections.
- Phoenix Score: the sum of the respiratory, cardiovascular, coagulation, and neurologic score.
- Sepsis: A pediatric patient with a suspected (or confirmed) infection and a Phoenix Score of at least two points is defined to have sepsis.
- Septic Shock: Sepsis with at least one cardiovascular point.

4.1 R

The inputs for the R function `phoenix` are the same as the inputs for the four individual organ dysfunction scores. The return form `phoenix` is a `data.frame` with a column for each of the four organ dysfunction scores, the Phoenix score, and indicators for sepsis and septic shock.

```r
phoenix_scores <-
  phoenix(
    # respiratory
      pf_ratio = pao2 / fio2,
      sf_ratio = ifelse(spo2 <= 97, spo2 / fio2, NA_real_),
      imv = vent,
      other_respiratory_support = as.integer(fio2 > 0.21),
    # cardiovascular
      vasoactives = dobutamine + dopamine + epinephrine + milrinone +
                    norepinephrine + vasopressin,
      lactate = lactate,
      age = age,
      map = dbp + (sbp - dbp)/3,
    # coagulation
      platelets = platelets,
      inr = inr,
      d_dimer = d_dimer,
      fibrinogen = fibrinogen,
    # neurologic
      gcs = gcs_total,
```

```r
      fixed_pupils = as.integer(pupil == "both-fixed"),
    data = sepsis
  )

str(phoenix_scores)
## 'data.frame':    20 obs. of  7 variables:
##  $ phoenix_respiratory_score   : int  0 3 3 0 0 3 3 0 3 3 ...
##  $ phoenix_cardiovascular_score: int  2 2 1 0 0 1 4 0 3 0 ...
##  $ phoenix_coagulation_score   : int  1 1 2 1 0 2 2 1 1 0 ...
##  $ phoenix_neurologic_score    : int  0 1 0 0 0 1 0 0 1 1 ...
##  $ phoenix_sepsis_score        : int  3 7 6 1 0 7 9 1 8 4 ...
##  $ phoenix_sepsis              : int  1 1 1 0 0 1 1 0 1 1 ...
##  $ phoenix_septic_shock        : int  1 1 1 0 0 1 1 0 1 0 ...
```

4.2 Python

```python
py_phoenix_scores = phx.phoenix(
    # resp
    pf_ratio = sepsis["pao2"] / sepsis["fio2"],
    sf_ratio = np.where(sepsis["spo2"] <= 97, sepsis["spo2"] / sepsis["fio2"], np.nan),
    imv      = sepsis["vent"],
    other_respiratory_support = (sepsis["fio2"] > 0.21).astype(int).to_numpy(),
    # cardio
    vasoactives = sepsis["dobutamine"] + sepsis["dopamine"] +
                  sepsis["epinephrine"] + sepsis["milrinone"] +
                  sepsis["norepinephrine"] + sepsis["vasopressin"],
    lactate = sepsis["lactate"],
    age = sepsis["age"],
    map = phx.map(sepsis["sbp"], sepsis["dbp"]),
    # coag
    platelets = sepsis['platelets'],
    inr = sepsis['inr'],
    d_dimer = sepsis['d_dimer'],
    fibrinogen = sepsis['fibrinogen'],
    # neuro
    gcs = sepsis["gcs_total"],
    fixed_pupils = (sepsis["pupil"] == "both-fixed").astype(int),
    )
print(py_phoenix_scores.info())
## <class 'pandas.core.frame.DataFrame'>
```

```
## RangeIndex: 20 entries, 0 to 19
## Data columns (total 7 columns):
##  #   Column                        Non-Null Count  Dtype
## ---  ------                        --------------  -----
##  0   phoenix_respiratory_score     20 non-null     int64
##  1   phoenix_cardiovascular_score  20 non-null     int64
##  2   phoenix_coagulation_score     20 non-null     int64
##  3   phoenix_neurologic_score      20 non-null     int64
##  4   phoenix_sepsis_score          20 non-null     int64
##  5   phoenix_sepsis                20 non-null     int64
##  6   phoenix_septic_shock          20 non-null     int64
## dtypes: int64(7)
## memory usage: 1.2 KB
## None
print(py_phoenix_scores.head())
##    phoenix_respiratory_score  ...  phoenix_septic_shock
## 0                          0  ...                     1
## 1                          3  ...                     1
## 2                          3  ...                     1
## 3                          0  ...                     0
## 4                          0  ...                     0
##
## [5 rows x 7 columns]
```

4.3 SQLite

```
CREATE TABLE IF NOT EXISTS phoenix AS

SELECT
  respiratory.pid AS pid,
  --phoenix_respiratory_score,
  --phoenix_cardiovascular_score,
  --phoenix_coagulation_score,
  --phoenix_neurologic_score,
  phoenix_respiratory_score + phoenix_cardiovascular_score +
    phoenix_coagulation_score + phoenix_neurologic_score AS phoenix_sepsis_score,

  IIF(phoenix_respiratory_score + phoenix_cardiovascular_score +
    phoenix_coagulation_score + phoenix_neurologic_score >=2, 1, 0) AS phoenix_sepsis,
```

```
  IIF(phoenix_respiratory_score + phoenix_cardiovascular_score +
    phoenix_coagulation_score + phoenix_neurologic_score >=2 AND
    phoenix_cardiovascular_score > 0, 1, 0) AS phoenix_septic_shock

FROM respiratory
LEFT JOIN cardiovascular
ON respiratory.pid = cardiovascular.pid
LEFT JOIN coagulation
ON respiratory.pid = coagulation.pid
LEFT JOIN neurologic
ON respiratory.pid = neurologic.pid
```

```
SELECT * FROM phoenix
```

Table 19: Displaying records 1 - 10

pid	phoenix_sepsis_score	phoenix_sepsis	phoenix_septic_shock
1	3	1	1
2	7	1	1
3	6	1	1
4	1	0	0
5	0	0	0
6	7	1	1
7	9	1	1
8	1	0	0
9	8	1	1
10	4	1	0

5 Phoenix-8

During development of the Phoenix criteria it was determined that the four-organ-system Phoenix criteria was sufficient for diagnosing sepsis. An extended eight-organ-system score, Phoenix-8, was defined and expected to be primarily used for research.

5.1 R

Calling to `phoenix8` within R will take the same inputs as the 8 organ dysfunction scoring functions. The return is the same as from `phoenix` with additional columns for each of the additional four organ systems and a Phoenix-8 total score.

```r
phoenix8_scores <-
  phoenix8(
    # respiratory
      pf_ratio = pao2 / fio2,
      sf_ratio = ifelse(spo2 <= 97, spo2 / fio2, NA_real_),
      imv = vent,
      other_respiratory_support = as.integer(fio2 > 0.21),
    # cardiovascular
      vasoactives = dobutamine + dopamine + epinephrine + milrinone +
                    norepinephrine + vasopressin,
      lactate = lactate,
      age = age, # Also used in the renal assessment.
      map = dbp + (sbp - dbp)/3,
    # coagulation
      platelets = platelets,
      inr = inr,
      d_dimer = d_dimer,
      fibrinogen = fibrinogen,
    # neurologic
      gcs = gcs_total,
      fixed_pupils = as.integer(pupil == "both-fixed"),
    # endocrine
      glucose = glucose,
    # immunologic
      anc = anc,
      alc = alc,
    # renal
      creatinine = creatinine,
      # no need to specify age again
    # hepatic
      bilirubin = bilirubin,
      alt = alt,
    data = sepsis
  )

str(phoenix8_scores)
## 'data.frame':    20 obs. of  12 variables:
##  $ phoenix_respiratory_score   : int  0 3 3 0 0 3 3 0 3 3 ...
##  $ phoenix_cardiovascular_score: int  2 2 1 0 0 1 4 0 3 0 ...
##  $ phoenix_coagulation_score   : int  1 1 2 1 0 2 2 1 1 0 ...
##  $ phoenix_neurologic_score    : int  0 1 0 0 0 1 0 0 1 1 ...
##  $ phoenix_sepsis_score        : int  3 7 6 1 0 7 9 1 8 4 ...
```

```
## $ phoenix_sepsis              : int  1 1 1 0 0 1 1 0 1 1 ...
## $ phoenix_septic_shock        : int  1 1 1 0 0 1 1 0 1 0 ...
## $ phoenix_endocrine_score     : int  0 0 0 0 0 0 0 0 1 0 ...
## $ phoenix_immunologic_score   : int  0 1 1 1 0 1 0 1 1 1 ...
## $ phoenix_renal_score         : int  1 0 0 0 0 1 1 0 1 0 ...
## $ phoenix_hepatic_score       : int  0 0 1 1 0 0 1 0 1 0 ...
## $ phoenix8_sepsis_score       : int  4 8 8 3 0 9 11 2 12 5 ...
```

5.2 Python

```
py_phoenix8_scores = phx.phoenix8(
  # resp
    pf_ratio = sepsis["pao2"] / sepsis["fio2"],
    sf_ratio = np.where(sepsis["spo2"] <= 97, sepsis["spo2"] / sepsis["fio2"], np.nan),
    imv      = sepsis["vent"],
    other_respiratory_support = (sepsis["fio2"] > 0.21).astype(int).to_numpy(),
  # card
    vasoactives = sepsis["dobutamine"] + sepsis["dopamine"] +
                  sepsis["epinephrine"] + sepsis["milrinone"] +
                  sepsis["norepinephrine"] + sepsis["vasopressin"],
    lactate = sepsis["lactate"],
    map = phx.map(sepsis["sbp"], sepsis["dbp"]),
    age = sepsis["age"], # also used in renal assessment
  # coag
    platelets = sepsis['platelets'],
    inr = sepsis['inr'],
    d_dimer = sepsis['d_dimer'],
    fibrinogen = sepsis['fibrinogen'],
  # neuro
    gcs = sepsis["gcs_total"],
    fixed_pupils = (sepsis["pupil"] == "both-fixed").astype(int),
  # endo
    glucose = sepsis["glucose"],
  # immuno
    anc = sepsis["anc"],
    alc = sepsis["alc"],
  # renal
    creatinine = sepsis["creatinine"],
    # no need to specify age again
  # hep
```

```
    bilirubin = sepsis["bilirubin"],
    alt = sepsis["alt"],
    )
print(py_phoenix8_scores.info())
## <class 'pandas.core.frame.DataFrame'>
## RangeIndex: 20 entries, 0 to 19
## Data columns (total 12 columns):
##  #   Column                         Non-Null Count  Dtype
## ---  ------                         --------------  -----
##  0   phoenix_respiratory_score     20 non-null     int64
##  1   phoenix_cardiovascular_score  20 non-null     int64
##  2   phoenix_coagulation_score     20 non-null     int64
##  3   phoenix_neurologic_score      20 non-null     int64
##  4   phoenix_sepsis_score          20 non-null     int64
##  5   phoenix_sepsis                20 non-null     int64
##  6   phoenix_septic_shock          20 non-null     int64
##  7   phoenix_endocrine_score       20 non-null     int64
##  8   phoenix_immunologic_score     20 non-null     int64
##  9   phoenix_renal_score           20 non-null     int64
##  10  phoenix_hepatic_score         20 non-null     int64
##  11  phoenix8_score                20 non-null     int64
## dtypes: int64(12)
## memory usage: 2.0 KB
## None
print(py_phoenix8_scores.head())
##    phoenix_respiratory_score  ...  phoenix8_score
## 0                          0  ...               4
## 1                          3  ...               8
## 2                          3  ...               8
## 3                          0  ...               3
## 4                          0  ...               0
##
## [5 rows x 12 columns]
```

5.3 SQLite

```
CREATE TABLE IF NOT EXISTS phoenix8 AS

SELECT
  respiratory.pid AS pid,
```

```sql
  --phoenix_respiratory_score,
  --phoenix_cardiovascular_score,
  --phoenix_coagulation_score,
  --phoenix_neurologic_score,

  phoenix_respiratory_score + phoenix_cardiovascular_score +
    phoenix_coagulation_score + phoenix_neurologic_score AS phoenix_sepsis_score,

  IIF(phoenix_respiratory_score + phoenix_cardiovascular_score +
    phoenix_coagulation_score + phoenix_neurologic_score >=2, 1, 0) AS phoenix_sepsis,

  IIF(phoenix_respiratory_score + phoenix_cardiovascular_score +
    phoenix_coagulation_score + phoenix_neurologic_score >=2 AND
    phoenix_cardiovascular_score > 0, 1, 0) AS phoenix_septic_shock,

  --phoenix_endocrine_score,
  --phoenix_immunologic_score,
  --phoenix_renal_score,
  --phoenix_hepatic_score,

  phoenix_respiratory_score + phoenix_cardiovascular_score +
    phoenix_coagulation_score + phoenix_neurologic_score  +
    phoenix_endocrine_score + phoenix_immunologic_score +
    phoenix_renal_score + phoenix_hepatic_score AS phoenix8_sepsis_score

FROM respiratory
LEFT JOIN cardiovascular
ON respiratory.pid = cardiovascular.pid
LEFT JOIN coagulation
ON respiratory.pid = coagulation.pid
LEFT JOIN neurologic
ON respiratory.pid = neurologic.pid
LEFT JOIN endocrine
ON respiratory.pid = endocrine.pid
LEFT JOIN immunologic
ON respiratory.pid = immunologic.pid
LEFT JOIN renal
ON respiratory.pid = renal.pid
LEFT JOIN hepatic
ON respiratory.pid = hepatic.pid
```

```
SELECT * FROM phoenix8
```

Table 20: Displaying records 1 - 10

pid	phoenix_sepsis_score	phoenix_sepsis	phoenix_septic_shock	phoenix8_sepsis_score
1	3	1	1	4
2	7	1	1	8
3	6	1	1	8
4	1	0	0	3
5	0	0	0	0
6	7	1	1	9
7	9	1	1	11
8	1	0	0	2
9	8	1	1	12
10	4	1	0	5

6 Clinical Vignettes

These are taken from the supplemental material of [8]

6.1 Clinical Vignette 1

A previously healthy 3-year-old girl presents to an emergency department in Lima, Peru, with a temperature of 39C, tachycardia, and irritability. Blood pressure with an oscillometric device is 67/32 mmHg (mean arterial pressure of 43 mmHg). She is given fluid resuscitation per local best practice guidelines, is started on broad spectrum antibiotics, and blood and urine cultures are sent. After an hour, she becomes hypotensive again and she is started on a norepinephrine drip. A complete blood count reveals leukocytosis, mild anemia, and a platelet count of 95 K/μL.

Phoenix Sepsis Score:

- 0 respiratory points (no hypoxemia or respiratory support)
- 2 cardiovascular points (1 for low mean arterial pressure for age, 1 for use of a vasoactive medication)
- 1 coagulation points (for low platelet count)
- 0 neurologic points (irritability would result in a Glasgow Coma Scale of approximately 14)
- total = 3 points.

Phoenix Sepsis Criteria: The patient has suspected infection, ≥ 2 points of the Phoenix Sepsis Score, and ≥ 1 cardiovascular points, so she meets criteria for septic shock.

```
# R
phoenix(
  vasoactives = 1,  # norepinephrine drip
  map = map(sbp = 67, dbp = 32), # 43.667 mmHg
  platelets = 95,
  gcs = 14, # irritability
  age = 3 * 12 # expected input for age is in months
  ) |>
str()
## 'data.frame':    1 obs. of  7 variables:
##  $ phoenix_respiratory_score   : int 0
##  $ phoenix_cardiovascular_score: int 2
##  $ phoenix_coagulation_score   : int 1
##  $ phoenix_neurologic_score    : int 0
##  $ phoenix_sepsis_score        : int 3
##  $ phoenix_sepsis              : int 1
##  $ phoenix_septic_shock        : int 1
```

```
# python
phx.phoenix(
  vasoactives = 1,  # norepinephrine drip
  map = phx.map(sbp = 67, dbp = 32), # 43.667 mmHg
  platelets = 95,
  gcs = 14, # irritability
  age = 3 * 12 # expected input for age is in months
  )
##    phoenix_respiratory_score  ...  phoenix_septic_shock
## 0                          0  ...                     1
##
## [1 rows x 7 columns]
```

6.2 Clinical Vignette 2

A 6-year-old boy with a history of prematurity presents with respiratory distress to his pediatrician's office in Tucson, Arizona. He is noted to have a temperature of 38.7C, tachypnea, crackles in the left lower quadrant on chest auscultation, and an oxygen saturation of 89% on room air. He is started on supplemental oxygen and is transported to the local emergency department via ambulance. In the emergency department, a chest X-ray shows a consolidation in the left lower lobe and hazy bilateral lung opacities, so he is started on antibiotics for

a suspected bacterial pneumonia. His respiratory status worsens, and he is started on non-invasive positive pressure ventilation. While awaiting to be admitted, his level of consciousness deteriorates rapidly: with nailbed pressure he only opens his eyes briefly, moans in pain, and withdraws his hand (Glasgow Coma Scale: 2 for eye response + 2 for verbal response + 4 for motor response = 8). He is intubated using rapid sequence induction and placed on a conventional ventilator. During this time, his lowest mean arterial pressure using a non-invasive oscillometric device is 52 mmHg and he receives a fluid bolus. He is then transferred to the pediatric intensive care unit where he requires a high positive end expiratory pressure and an FiO_2 of 0.45 to achieve an oxygen saturation of 92% (S/F ratio: 204). Complete blood count and lactate level reveal a platelet count of 120 K/μL and a serum lactate of 2.9 mmol/L. Given his platelet count below the normal reference range, a coagulation panel is sent, which reveals an INR of 1.7, a D-Dimer of 4.4 mg/L, and a fibrinogen of 120 mg/dL.

Phoenix Sepsis Score:

- 2 respiratory points (for an S/F ratio < 292 on invasive mechanical ventilator)
- 0 cardiovascular points (mean arterial pressure > 48 mmHg and Lactate level < 5 mmol/L)
- 2 coagulation points (for high INR and D-Dimer)
- 1 neurologic point (Glasgow Coma Scale < 10)
- total = 5 points.

Phoenix Sepsis Criteria: The patient has a suspected infection, ≥ 2 points of the Phoenix Sepsis Score, and 0 cardiovascular points, so he meets criteria for sepsis.

```
# R
phoenix(
  gcs = 2 + 2 + 4, # eye + verbal + motor
  map = 52,
  imv = 1,
  sf_ratio = 92 / 0.45,
  platelets = 120,
  lactate = 2.9,
  inr = 1.7,
  d_dimer = 4.4,
  fibrinogen = 120)
##   phoenix_respiratory_score phoenix_cardiovascular_score
## 1                         2                            0
##   phoenix_coagulation_score phoenix_neurologic_score phoenix_sepsis_score
## 1                         2                        1                    5
##   phoenix_sepsis phoenix_septic_shock
## 1              1                    0
```

```
# Python
phx.phoenix(
  gcs = 2 + 2 + 4, # eye + verbal + motor
  map = 52,
  imv = 1,
  sf_ratio = 92 / 0.45,
  platelets = 120,
  lactate = 2.9,
  inr = 1.7,
  d_dimer = 4.4,
  fibrinogen = 120)
##    phoenix_respiratory_score  ...  phoenix_septic_shock
## 0                          2  ...                     0
##
## [1 rows x 7 columns]
```

References

1 Csárdi G, Hester J, Wickham H, *et al.* *Remotes: R package installation from remote repositories, including 'GitHub'*. 2024.

2 Matics TJ, Sanchez-Pinto LN. Adaptation and Validation of a Pediatric Sequential Organ Failure Assessment Score and Evaluation of the Sepsis-3 Definitions in Critically Ill Children. *JAMA Pediatrics*. 2017;171:e172352–2. doi: 10.1001/jamapediatrics.2017.2352

3 Gaies MG, Gurney JG, Yen AH, *et al.* Vasoactive–inotropic score as a predictor of morbidity and mortality in infants after cardiopulmonary bypass. *Pediatric critical care medicine*. 2010;11:234–8.

4 Dewi W, Christie C, Wardhana A, *et al.* Pediatric logistic organ dysfunction-2 (pelod-2) score as a model for predicting mortality in pediatric burn injury. *Annals of Burns and Fire Disasters*. 2019;32:135.

5 Khemani RG, Bart RD, Alonzo TA, *et al.* Disseminated intravascular coagulation score is associated with mortality for children with shock. *Intensive care medicine*. 2009;35:327–33.

6 Bembea MM, Agus M, Akcan-Arikan A, *et al.* Pediatric organ dysfunction information update mandate (PODIUM) contemporary organ dysfunction criteria: Executive summary. *Pediatrics.* 2022;149:S1–12.

7 Goldstein B, Giroir B, Randolph A, *et al.* International pediatric sepsis consensus conference: Definitions for sepsis and organ dysfunction in pediatrics. *Pediatric critical care medicine.* 2005;6:2–8.

8 Sanchez-Pinto LN, Bennett TD, DeWitt PE, *et al.* Development and Validation of the Phoenix Criteria for Pediatric Sepsis and Septic Shock. *JAMA.* 2024;331:675–86. doi: 10.1001/jama.2024.0196
